# A 7 gene expression score predicts for radiation response in cancer cervix

**DOI:** 10.1186/1471-2407-9-365

**Published:** 2009-10-15

**Authors:** Thangarajan Rajkumar, Neelakantan Vijayalakshmi, Kesavan Sabitha, Sundersingh Shirley, Ganesharaja Selvaluxmy, Mayil Vahanan Bose, Lavanya Nambaru

**Affiliations:** 1 Department of Molecular Oncology, Cancer Institute (WIA), Chennai, India; 2 Department of Pathology, Cancer Institute (WIA), Chennai, India; 3 Department of Radiation Oncology, Cancer Institute (WIA), Chennai, India

## Abstract

**Background:**

Cervical cancer is the most common cancer among Indian women. The current recommendations are to treat the stage IIB, IIIA, IIIB and IVA with radical radiotherapy and weekly cisplatin based chemotherapy. However, Radiotherapy alone can help cure more than 60% of stage IIB and up to 40% of stage IIIB patients.

**Methods:**

Archival RNA samples from 15 patients who had achieved complete remission and stayed disease free for more than 36 months (No Evidence of Disease or NED group) and 10 patients who had failed radical radiotherapy (Failed group) were included in the study. The RNA were amplified, labelled and hybridized to Stanford microarray chips and analyzed using BRB Array Tools software and Significance Analysis of Microarray (SAM) analysis. 20 genes were selected for further validation using Relative Quantitation (RQ) Taqman assay in a Taqman Low-Density Array (TLDA) format. The RQ value was calculated, using each of the NED sample once as a calibrator. A scoring system was developed based on the RQ value for the genes.

**Results:**

Using a seven gene based scoring system, it was possible to distinguish between the tumours which were likely to respond to the radiotherapy and those likely to fail. The mean score ± 2 SE (standard error of mean) was used and at a cut-off score of greater than 5.60, the sensitivity, specificity, Positive predictive value (PPV) and Negative predictive value (NPV) were 0.64, 1.0, 1.0, 0.67, respectively, for the low risk group.

**Conclusion:**

We have identified a 7 gene signature which could help identify patients with cervical cancer who can be treated with radiotherapy alone. However, this needs to be validated in a larger patient population.

## Background

Cervical cancer is a preventable but the most common cancer among Indian women and second most common cancer among women worldwide [1,2]. More than 500,000 women are expected to develop the disease every year worldwide. The tragedy is worsened by most of the women presenting in locally advanced disease as well. Human Papilloma virus has been identified as an important factor in the development of cervical cancer, but it alone is not sufficient and requires additional events [3]. Cervical cancer progresses through initial dysplastic or pre-malignant stages before becoming invasive. The evolution to an invasive cancer may take up to 15 years or more [4]. PAP smear testing has been found to aid in early detection of pre-malignant lesions, thereby preventing the morbidity and mortality of cervical cancer. However, organized screening is primarily available in the Western countries and most of the developing countries including India do not have an organized screening program.

The standard treatment currently recommended for stage IIB, IIIA, IIIB and IVA has been concurrent chemo-radiotherapy, usually with weekly cisplatin based chemotherapy [5]. This is based on the data from 5 randomized trials which had shown a 30 -50% benefit in reducing the risk of death compared to the radiotherapy only arm. While the addition of chemotherapy is clearly beneficial, not all patients may need it. Currently, there are no reliable ways of identifying the individuals who may respond to the radical radiotherapy from those who will not respond.

The use of molecular approaches, such as gene expression studies, has helped tailor treatment based on the molecular characteristics. The Oncotype Dx testing for breast cancer is an example of using the newer techniques to help tailor the treatment to individual patient [6]. Several potential markers for tumour response have been identified in the past, such as c-myc, Bcl2, Bax, HIF2α, Ki67 index, c-erbB2 [7-11]. However, most of these studies were on smaller number of patients and have not been used routinely to predict radiation response. More recently a few studies have used microarray based technology to identify gene signatures predictive of radiation response [12-14] however, none of them had validated their genes using further techniques such as Real time Polymerase Chain Reaction (PCR).

Avoiding morbidity and the cost of chemo-radiation in cervical cancer would be relevant particularly in a developing country. This study aims to develop a test which can help identify patients who may not need chemotherapy and who can be cured only with radical radiotherapy.

## Methods

Archival total RNA extracted from punch biopsy samples collected in RNA later (Ambion, Austin, Tx; Cat no: AM7021) and stored in the tumour bank after an informed consent were used, after obtaining the Institutional Ethical committee's approval for the study. The RNA had been extracted from the biopsy samples using the RNeasy RNA extraction kit (Qiagen, Gmbh, Hilden; Cat no: 74106) as per the manufacturer's instructions.

Twenty five patients' samples were included in the study. The criteria for inclusion in the study were as follows: 1. good quality RNA as assessed by Bio-analyser (RIN 6 or above); 2. paired paraffin block having at least 70% tumor cells; 3. sufficient quantity of RNA be available; 4. patient should have completed prescribed radiotherapy and follow-up information till death/last disease free status be available.

All the patients had received radical radiotherapy, with 6 MV X-ray from Linear accelerator and brachytherapy with HDR-Ir^192^. The total dose given was between 64 - 66 Gy, over 56 - 60 days. The median and mean follow-up period among the NED group was 43 months and 42 months, respectively, with a minimum and maximum period of follow up of 38 and 45 months, respectively. All the Failed patients had been followed up till progression/relapse/death. Complete remission refers to complete resolution of measurable disease by clinical examination, hematologic, biochemical or radiological examination; Partial remission refers to 50% or more disease reduction lasting longer than one month; Progression refers to worsening or advancing disease.

### HPV testing

The quality of the DNA was assessed by amplifying for β globin prior to HPV testing which was done using GP5+ and GP6+ primers [15]. HPV16 and 18 typing was done using Nested Multiplex Polymerase Chain Reaction (NMPCR) technique [16]. SiHa DNA for HPV16, HeLa DNA for HPV18 (positive controls) and C33A DNA (negative control) were included in all runs.

### Microarray experiment

1 μg of total RNA from the tumour sample and universal RNA (Stratagene; Cat no: 740000-41) were reverse transcribed using Array script at 42°C for 2 hrs to obtain cDNA using the Amino Allyl MessageAmp II aRNA amplification kit (Ambion, Austin, Tx; Cat no: AM1797). The cDNA was amplified by in-vitro transcription in the presence of T7 RNA polymerase; aRNA thus obtained was purified and quantitated in NanoDrop (NanoDrop Technologies, Wilmington, DE, USA). 20 μg of tumour aRNA was labelled using NHS ester of Cy5 dye and control universal aRNA was labelled using NHS ester of Cy3 dye. The Cy3 and Cy5 labelled aRNA was used for hybridization onto the microarray chips from Stanford Functional Genomics Facility (SFGF, Stanford, CA) containing 44,544 spots, for 16 hrs in Lucidea SlidePro hybridization chamber (GE Health Care, Uppsala, Sweden) at 42°C. After hybridization, slides were washed in 0.1× SSC, 1× SSC followed by 0.1× SSC and dried.

The slides were scanned in ProScanArray (PerkinElmer, Shelton, CT, USA). Griding was done using Scan array Express software package (version -4). The integrated or mean intensity of signal within the spot was calculated. The files were saved as GPR files.

**All the raw data files have been submitted to GEO with an assigned GEO accession number - GSE14404**.

### Microarray data analysis

The Foreground Median intensity for Cy3 and Cy5, Background Median intensity for Cy3 and Cy5, spot size data were imported into BRB-ArrayTools software [17] using the Import wizard function. Background correction was not done. Global normalization was used to median centre the log-ratios on each array in order to adjust for differences in labelling intensities of the Cy3 and Cy5 dyes. The data was analysed using the Class comparison module in the BRB-Array Tools software. The normalized Log ratios were also imported into Significance Analysis of Microarray (SAM) [18] software and analysed.

### Class Comparison in BRB-Array Tools

We identified genes that were differentially expressed among the two classes (NED VS Failed) using a random-variance t-test. The random-variance t-test is an improvement over the standard separate t-test as it permits sharing information among genes about within-class variation without assuming that all genes have the same variance [19]. Genes were considered statistically significant if their p value was < 0.0015. In addition a two fold difference was required between the two classes.

### SAM Analysis

The normalized log ratios of all the samples were imported into SAM software and analysed. First a two class unpaired analysis with 100 permutations was done. A delta value of 0.96 and a fold difference of 2 was used to identify the genes differentially expressed. In addition, Survival analysis for significant genes was done using 100 permutations and with a delta of 0.85.

### Quantitative Real time PCR

High Capacity Reverse Transcription kit (Applied Biosystems, Foster City, CA; Cat no: 4368814) was used to reverse transcribe 2 μg of total RNA from the 23 samples in a 20 μl reaction volume. In two samples, due to the limiting amount of RNA, 0.75 μg was used for the cDNA synthesis.

These cDNA samples were used for real time PCR amplification assays using TaqMan^® ^arrays formerly TaqMan^® ^Low density arrays (TLDA) (Applied Biosystems, Foster City, CA; Cat no: 4342261). The fluorogenic, FAM labelled probes and the sequence specific primers for the list of genes with endogenous control 18S rRNA were obtained as inventoried assays and incorporated into the TaqMan^® ^array format. Quadruplicate (n = 23) and duplicate (n = 2; with limiting amount of RNA for cDNA synthesis) cDNA template samples were amplified and analysed on the ABI Prism 7900HT sequence detection system (Applied Biosystems, Foster City, CA).

The reaction set up, briefly, consisted of 1.44 μg of cDNA template made up to 400 μl with deionised water and equal amounts of TaqMan^® ^Universal PCR Master Mix (Applied Biosystems, Foster City, CA; Cat no: 4304437). 100 μl was loaded into each of the 8 ports of the array (2 ports comprise of one sample replicate on the array). Thus, the samples run as duplicates were only loaded into 4 ports of the array. Thermal cycling conditions included a 50°C step for 2 minutes, denaturation for 10 min at 94°C followed by 40 cycles consisting of 2 steps: 97°C for 30 seconds and 59.7°C for 1 minute for annealing and extension.

The raw data from the Prism 7900HT sequence detection system was imported into the Real-Time StatMiner™ software for statistical analysis of the data. Among the endogenous reference genes included on the array (18S ribosomal gene; UBC, β2 microglobulin), UBC was chosen after visualizing the global Ct value distribution, for normalizing the data (see Additional file 1). The TLDA assays were run at LabIndia Instruments Pvt Ltd laboratories at Gurgaon, New Delhi.

### Development of the scoring method

Relative quantification is based on the relative expression of a target gene versus a reference gene. The UBC normalized Delta Ct values were imported into the Excel spread sheet for obtaining the RQ values for the 24 samples (one sample which had not worked in RQ-RT-PCR was excluded), using each NED sample once as a calibrator. The genes were provided a score of 2, if the RQ value was >2. This analysis was done initially on all the selected genes taken up for validation and then on genes which had greater than two fold difference between the two classes, with an attempt to obtain a group of genes which can help distinguish between the two classes. The Mean score, the standard error (SE), the mean score ± 1SE and mean score ± 2SE were calculated and the Mean ± 2SE was used for categorizing as either High risk or Low risk. The cut-off was set at 40% (5.6) of the potential total score possible.

Sensitivity is the probability for a NED sample to be correctly predicted as Low risk; Specificity is the probability for a Failed sample to be correctly predicted as High risk; PPV is the probability that a sample predicted as Low risk actually belongs to class NED; NPV is the probability that a sample predicted as High risk actually does not belong to NED [17].

### Statistical Analysis

Kaplan Meier survival analysis was used to study the disease free survival (DFS) in the two classes, using the Stata 10 software. This was done initially based on the stage of the disease and then based on the gene signature risk stratification (Score ≤ 5.60 and score >5.60). Log rank test was used to assess the survival difference.

## Results

The patients' clinico-pathological status and their response to treatment are given in Table 1. Twenty four of the tumours were Squamous cell carcinomas (16 Large cell non-keratinizing, 4 large cell keratinizing and 4 unspecified) and one was a poorly differentiated carcinoma. Fourteen were HPV16 positive, 7 were HPV18 positive and 4 were HPV16 and 18 subtype negative (but HPV positive).

**Table 1:** Clinico-pathological and disease free status of the patients

ID	Stage	Grade	HPE	Subtype	HPV Subtype	Treatment given	Response	Outcome (NED/Failed)	Site of failure	DFS (months)	OS (months)
hoae145	IIB	III	SCC	LCNK	16	RT	CR	NED		44	44
hoae146	IIB	III	SCC	LCNK	Negative	RT	CR	NED		38	38
hoae152	IIIB	III	SCC	LCNK	18	RT	CR	NED		45	45
hoae153	IIB	III	SCC	LCK	16	RT	CR	NED		45	45
hov156	IIB	III	SCC	LCNK	18	RT	CR	NED		43	43
HOV160	IIB	III	SCC	LCNK	16	RT	CR	NED		44	44
HOV161	IIB	III	SCC	LCNK	18	RT	CR	NED		41	41
HOV164	IIIB	III	SCC	LCNK	Negative	RT	CR	NED		38	38
hov165	IIB	III	SCC	Not specified	16	RT	CR	NED		43	43
hov182	IIA	III	SCC	LCNK	16	RT	CR	NED		39	39
hov183	IIB	III	SCC	LCNK	18	RT	CR	NED		45	45
hov189	IIA	III	SCC	LCNK	16	RT	CR	NED		40	40
hov190	IB	III	SCC	LCNK	16	RT	CR	NED		42	42
hov196*	IIB	II-III	SCC	LCNK	16	RT+CT	PR	Failed	Local	0	6
hov198	IIA	III	SCC	LCNK	16	RT	PR	Failed	Local	0	13
hov199	IIB	III	SCC	LCNK	18	RT	CR	Failed	Distant	10	34
hov200	IIIB	II-III	SCC	LCK	16	RT	PR	Failed	Local	0	24
hov217	IIB	III	SCC	LCNK	18	RT	PR	Failed	Local	0	7
hov218	IIB	III	SCC	LCNK	Negative	RT	CR	Failed	Distant	18	23
hov220	IIB	III	SCC	Not specified	16	RT	PR	Failed	Local	0	11
hov232	IIA	III	SCC	Not specified	16	RT	CR	NED		46	46
hov240	IIB	III	SCC	LCNK	16	RT	CR	NED		38	38
HOV241	IIB	III	PD	Not specified	Negative	RT	PR	Failed	Local	0	12
HOV242	IIIB	II	SCC	LCK	16	RT	PR	Failed	Local	0	15
hov247	IIB	III	SCC	LCK	18	RT	PR	Failed	Local	0	7

* - One patient had received concurrent chemo-radiotherapy and had failed, was also included in the study

NED = No evidence of disease; SCC = Squamous cell carcinoma; PD = Poorly differentiated; LCNK = Large cell non-keratinizing;

LCK = Large cell keratinizing; CR = Complete remission; PR = Partial remission; RT = Radiotherapy; CT = Chemotherapy

Using different methods, as described above, genes that were found to be differentially expressed between the two classes (NED and Failed) were obtained. The List of genes significant by different methods of analysis are given as a Additional file 2.

Twenty genes were selected for further validation by RQ-PCR using the Taqman Low Density Array card (TLDA) format (Table 2). These 20 genes formed part of the 95 genes selected for analysis using the TLDA format. Most of the additional genes were those which had been found to be differentially expressed between cervical cancer and normal cervix; cervical cancer and CIN1; CIN3 and CIN1; CIN3 and Normal cervix. Apart from the mandatory endogenous 18S rRNA included in the TLDA cards, based on the microarray data, UBC and β2 microglobulin, were included as additional endogenous reference genes.

**Table 2:** List of genes selected for RQ-RT-PCR validation

GENE SYMBOL	GENE NAME	ASSAY ID	AMPLICON SIZE
TRAPPC6A	trafficking protein particle complex 6A	Hs00225487_m1	133
FLJ90231	Galactosidase, beta 1-like 3	Hs00293572_m1	109
MRCL3	myosin regulatory light chain MRCL3	Hs00272402_m1	105
ASB16	ankyrin repeat and SOCS box-containing 16	Hs00365575_m1	79
C11orf9	chromosome 11 open reading frame 9	Hs00203205_m1	56
PLA2G6	phospholipase A2, group VI (cytosolic, calcium-independent)	Hs00185926_m1	89
YAP1	Yes-associated protein 1, 65 kDa	Hs00371735_m1	64
BIRC2	baculoviral IAP repeat-containing 2	Hs01112284_m1	84
MMP3	matrix metallopeptidase 3 (stromelysin 1, progelatinase)	Hs00968308_m1	98
FST	follistatin	Hs00246260_m1	105
DCUN1D5	DCN1, defective in cullin neddylation 1, domain containing 5 (S. cerevisiae)	Hs00260376_m1	61
HCRTR1	hypocretin (orexin) receptor 1	Hs00173513_m1	78
TMEM123	transmembrane protein 123	Hs00364685_m1	56
ASPN	asporin	Hs00214395_m1	72
CCL18	chemokine (C-C motif) ligand 18 (pulmonary and activation-regulated)	Hs00268113_m1	83
CTSC	cathepsin C	Hs00175188_m1	71
SDCCAG8	serologically defined colon cancer antigen 8	Hs00394864_m1	79
THOC6	THO complex 6 homolog (Drosophila)	Hs00738020_g1	67
CES2	carboxylesterase 2 (intestine, liver)	Hs00187279_m1	88
OPTC	opticin	Hs00205039_m1	71
***IGF2BP2***	***insulin-like growth factor 2 mRNA binding protein 2***	***Hs01118009_m1***	***80***
***UBE2C***	***ubiquitin-conjugating enzyme E2C***	***Hs00738962_m1***	***91***

The last 2 genes were found to be overexpressed in Failed to compared to NED samples, although they had been included initially as genes differentially expressed between the tumours/CIN3 and normal cervix.

One of the sample CXL19-hov160 which had worked in microarray did not amplify in the RQ-TLDA assay and had to be removed from further analysis. In addition, OPTC and C11orf9 genes did not amplify in any of the samples.

The RQ values after calibrating with the NED samples (Mean) for all the 95 genes showed two additional genes, UBE2C and IGF2BP2, to be overexpressed in the Failed samples compared to the NED samples. These two genes had initially been chosen for validation of the differentially expressed genes between cancer and normal cervix. After excluding the genes which did not amplify, we now had 20 genes for further analysis (see Additional file 3).

There were 10 genes (including UBE2C and IGF2BP2) with more than 2 fold difference between the two classes. In addition, 4 genes had a fold change between 1 and 2. There was only 44% (8/18) concordance between the microarray data and the Relative quantitation RT-PCR data, when a 2 fold difference was used as the criteria. The best concordant rates were with the SAM survival analysis, wherein of the 11 genes taken up for validation, 8 were concordant (73%).

We then tried to analyse the RQ data using each of the NED sample once as a calibrator. The focus was on genes which had an over-expression of at least two fold between the two classes. A scoring system was then developed as described in the Methods section. Using this we found that the seven gene score (UBE2C, MMP3, DCUN1D5, SDCCAG8, IGF2BP2, CCL18 and FST) can help distinguish between the Failed and the NED samples. Table 3 gives the mean score of the 24 experiments, the standard deviation, the standard error (SE), and score ± 2SE (see Additional file 4 for the details). Using a cut-off value of > 5.60, the sensitivity, specificity, positive predictive value (PPV) and negative predictive value (NPV) are 0.64, 1.0, 1.0, 0.67, respectively, for the Mean ± 2SE. At this cut-off point, 9 of the 14 (64) NED samples were classified as low risk, while all the failed samples were classified as high risk.

**Table 3:** Scores based on RQ-RT-PCR values

	MEAN SCORE	STDEV	SE	SCORE+2SE	SCORE-2SE
**DCt.CXL103-HOV220**	**9.57**	**1.95**	**0.52**	**10.61**	**8.53**
**DCt.CXL108-HOV199**	**7.86**	**1.99**	**0.53**	**8.92**	**6.79**
**DCt.CXL120-HOV241**	**12.86**	**1.51**	**0.4**	**13.67**	**12.05**
**DCt.CXL121-HOV247**	**10**	**1.36**	**0.36**	**10.73**	**9.27**
**DCt.CXL17-HOV200**	**7.86**	**2.66**	**0.71**	**9.28**	**6.44**
**DCt.CXL46-HOV198**	**7.43**	**2.98**	**0.8**	**9.02**	**5.84**
**DCt.CXL86-HOV218**	**6.86**	**2.32**	**0.62**	**8.09**	**5.62**
**DCt.CXL90-HOV217**	**7.14**	**2.57**	**0.69**	**8.52**	**5.77**
**DCt.CXL95-HOV242**	**13.14**	**1.29**	**0.35**	**13.83**	**12.45**
**DCt.CXM018-HOV196**	**8.86**	**1.7**	**0.46**	**9.77**	**7.95**
DCt.CXL111-HOV240	1.14	1.29	0.35	1.83	0.45
DCt.CXL13-HOV183	3.86	2.14	0.57	5	2.71
DCt.CXL14-HOV164	1.86	1.83	0.49	2.84	0.88
DCt.CXL16-HOV232	4.43	2.38	0.64	5.7	3.16
DCt.CXL20-HOV182	4.29	2.46	0.66	5.6	2.97
DCt.CXL22HOV161	3.71	2.7	0.72	5.16	2.27
DCt.CXL3-HOV165	5.57	2.85	0.76	7.09	4.05
DCt.CXL35-HOAE145	1.29	1.86	0.5	2.28	0.29
DCt.CXL52-HOAE152	4.29	1.9	0.51	5.3	3.27
DCt.CXL62-HOAE153	1.43	1.65	0.44	2.31	0.55
DCt.CXL73-HOAE146	3.43	2.98	0.8	5.02	1.84
DCt.CXL82-HOV189	4.86	2.32	0.62	6.09	3.62
DCt.CXL9-HOV156	4.57	3.18	0.85	6.27	2.87
DCt.CXL91-HOV190	2.57	1.99	0.53	3.63	1.51

Samples in bold are the failed samples;

STDEV = Standard deviation; SE = Standard error

**Figure 1:**
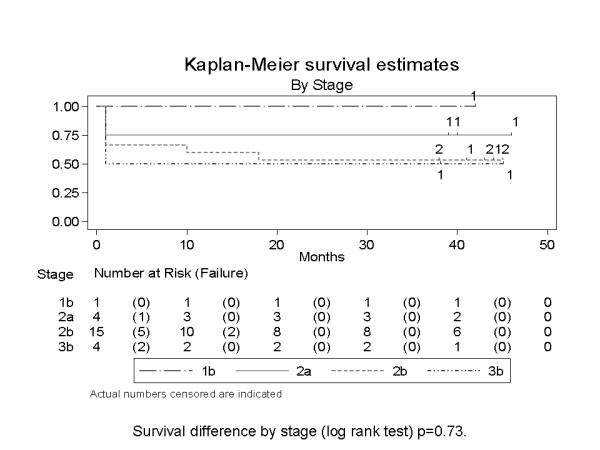
**Based on stage of disease**.

**Figure 2:**
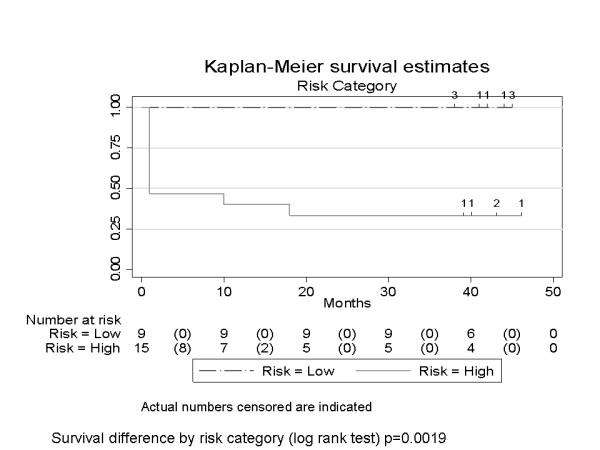
**(1) Kaplan Meier disease free survival analysis based on risk stratification (Low versus High risk) (2)**.

The Kaplan Meier survival curves are given in Figures 1 and 2, the former based on the stage of the disease and the latter based on the gene expression signature.

## Discussion

Cervical cancer although a preventable disease, still kills a significant number of women. Most of the tertiary centres in developing countries still see patients in advanced stages. Radiotherapy has been the main stay of treatment and the addition of concurrent cisplatin based chemotherapy has been found to improve cure rates. However, not all patients will need concurrent chemotherapy, which has its own cost implications and morbidity, particularly in a developing country. With the emphasis shifting to tailoring therapy to individuals based on the patient and tumour characteristics, it is essential to develop newer tests which can help identify individuals who can be cured only with radiotherapy. This paper provides one such approach, wherein a seven gene score was found to help distinguish those who are likely to be cured with radiotherapy alone from those who may require additional forms of treatment.

The concordance between the microarray and the RQ-PCR data on genes differentially expressed with more than 2 fold was 44% (8/18). SAM Survival analysis had identified 13 genes (of the 14 genes, one was a duplicate -YAP1) as being differentially expressed and 11 of these genes were included in our validation. One gene did not have a suitable assay for the TLDA format. Of the 11 genes tested, 8 were found to be concordant in the RQ-RT-PCR validation (73% concordance rate). The SAM Survival analysis had correctly identified 5 of the 7 genes which we had used for the prediction.

One group had addressed the issue of concordance, when they compared Affymetrix data with Real time quantitative data for 48 genes. Their concordance rate was around 69% and they have reported that this may be usually due to differences in the target transcripts being identified by the two platforms (microarray versus Real time PCR) [20].

Klopp et al [14] had used microarray analysis on samples obtained prior to treatment and 48 hours after start of chemoradiation in 12 patients and found that a 58 gene signature can help predict recurrence. Others have developed either a Lymph node prediction model using 156 gene signatures, which had a prediction accuracy of 77% in predicting lymph node metastasis [21] or thermo-radio-response prediction model using a 35 gene signature [22]. Wong et al [13] used supervised clustering analysis to classify radiosensitive and radioresistant tumours in 13 patients.

Six of the seven genes, identified in our study, are known to be involved in cancers, with some of them having a role in inducing therapeutic resistance.

### Insulin like growth factor 2 mRNA binding protein 2/IGF2BP2/IMP2

This oncofetal protein functions by binding to the 5'UTR region of IGF2 and thereby regulating its translation. Yisraeli (2005) [23] had suggested that the VICKZ family, to which IGF2BP2/IMP2 belongs, have a role to play in cell polarity and migration, cell proliferation and cancer. Autoantibodies to IGF2BP2 has been detected in different cancers including hepatocellular carcinoma [24,25]. IGF2BP2 was also found to be overexpressed in epithelial ovarian cancers [26].

### Ubiquitin-conjugating enzyme E2C/UBE2C/UBCH10

The gene is a member of the E2 ubiquitin-conjugating enzyme family, playing key roles in regulation of cell cycle. The gene was found to be overexpressed in cervical cancers [27] and was reported to contribute to chemotherapy resistance in breast cancer [28]. Donata et al [29] have reported over-expression in low grade astrocytomas and in glioblastomas, wherein they could contribute to therapeutic resistance.

### Follistatin/FST

This protein inhibits follicle stimulating hormone release. FST has been shown to be associated with Basal cell carcinomas [30]. In addition, integrin alpha6beta4, which is involved in apoptosis resistance, invasion, metastasis, can induce the expression of several genes, including FST, which could help mediate these processes [31].

### Matrix metallopeptidase 3 (stromelysin1; progelatinase)/MMP3

MMP3 has been reported to be expressed in lymph node metastasis and in recurrent cervical cancers [32]. MMP3 has been suggested to have a role in modulating chemotherapeutic response in head and neck cancers [33] and has been shown to play a role in breast tumour carcinogenesis [34].

### Chemokine (C-C motif) ligand 18/CCL18/MIP4/AMAC/DCCK1/PARC

CCL18/PARC has been found to be overexpressed in the serum of acute lymphoblastic leukaemia patients [35] and in the ascitic fluid of patients with ovarian carcinoma [36].

Serologically defined colon cancer antigen 8/SDCCAG8/Centrosomal colon cancer autoantigen protein/CCCAP

Autologous antibody responses to SDCCAG8 has been reported in colon and ovarian cancers [37,38]. It is a protein integral to the centrosomes [39].

### Defective in cullin neddylation 1, domain containing 5/DCUN1D5

Little is known about the function of this gene, particularly in cancer.

## Conclusion

Locally advanced cancer of the cervix is now being treated with chemo-radiotherapy. However, nearly 60% of stage IIB and 40% of stage IIIB can be cured with radiotherapy only. Currently there are no reliable ways of identifying those patients who can be cured with radiotherapy alone.

Using microarray technique followed by Relative Quantitation Real Time PCR (RQ RT-PCR), we have identified a 7 gene signature with differential expression between cervical cancers with and without recurrence/progression after radiation treatment. This finding needs to be validated in an independent larger patient population.

## Conflict of interests

The authors declare that they have no competing interests.

## Authors' contributions

TR conceived the study; acquired, analysed & interpreted the data and drafted and revised the article. NV standardized and performed the microarray experiments and was involved in the drafting of the article. KS was involved in the acquisition and analysis of the microarray data and in the drafting of the article. SS carried out all the pathological studies and assessment of samples for the microarray studies. GS was involved in the clinical management and data analysis and follow-up of the patients. MB performed the microarray experiments. LN was involved in sample collection and processing and analysis. All the authors read and approved the final version of the manuscript.

## Pre-publication history

The pre-publication history for this paper can be accessed here:

http://www.biomedcentral.com/1471-2407/9/365/prepub

## Supplementary Material

Additional file 1 **Ct values for reference genes**. Provides the Ct values of the reference genes. Click here for file

Additional file 2 **Genes significant by different methods of analysis of the microarray data**. The data shows the list of genes identified to be significant by different methods of the microarray analysis. Click here for file

Additional file 3 **RQ analysis of NED and Failed samples for the 22 genes**. The file provides the list of genes used for predictive analysis. Click here for file

Additional file 4 **Total scores derived from the scoring method**. The file provides details on the scoring of the samples. Click here for file
