# Supplementary figures and images for: A 7 gene expression score predicts for radiation response in cancer cervix
Source: BMC Cancer. 2009 Oct 15;9:365. doi: 10.1186/1471-2407-9-365 (PMC2768747; doi:10.1186/1471-2407-9-365)

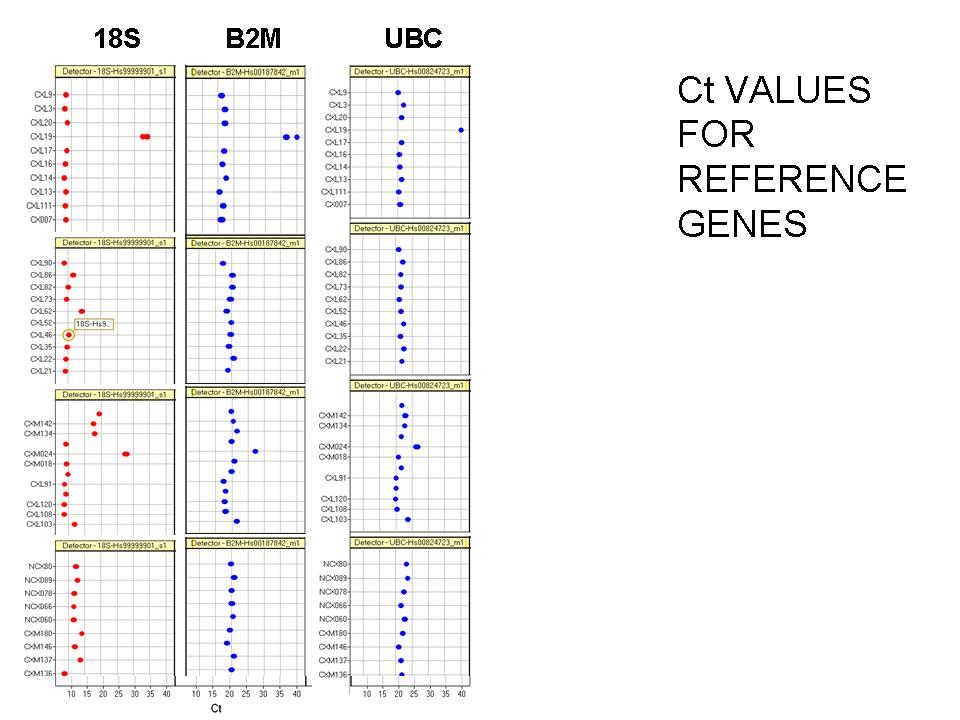

Supplement: Additional file 1 — Ct values for reference genes. Provides the Ct values of the reference genes. [file 1471-2407-9-365-S1.JPEG]
